# Influence of 5-N-carboxamide modifications on the thermodynamic stability of oligonucleotides

**DOI:** 10.1093/nar/gkv981

**Published:** 2015-10-04

**Authors:** Steven K. Wolk, Richard K. Shoemaker, Wes S. Mayfield, Andrew L. Mestdagh, Nebojsa Janjic

**Affiliations:** 1 SomaLogic, Inc., Boulder, CO 80301, USA; 2 Department of Chemistry and Biochemistry, University of Colorado, Boulder, CO 80309-0215, USA

## Abstract

We have recently shown that the incorporation of modified nucleotides such as 5-N-carboxamide-deoxyuridines into random nucleic acid libraries improves success rates in SELEX experiments and facilitates the identification of ligands with slow off-rates. Here we report the impact of these modifications on the thermodynamic stability of both duplexes and intramolecular ‘single-stranded’ structures. Within duplexes, large, hydrophobic naphthyl groups were destabilizing relative to the all natural DNA duplex, while the hydrophilic groups exhibited somewhat improved duplex stability. All of the significant changes in stability were driven by opposing contributions from the enthalpic and entropic terms. In contrast, both benzyl and naphthyl modifications stabilized intramolecular single-stranded structures relative to their natural DNA analogs, consistent with the notion that intramolecular folding allows formation of novel, stabilizing hydrophobic interactions. Imino proton NMR data provided evidence that elements of the folded structure form at temperatures well below the T_m_, with a melting transition that is distinctly less cooperative when compared to duplex DNA. Although there are no data to suggest that the unmodified DNA sequences fold into structures similar to their modified analogs, this still represents clear evidence that these modifications impart thermodynamic stability to the folded structure not achievable with unmodified DNA.

## INTRODUCTION

Since its inception in 1990, SELEX (Systematic Evolution of Ligands by EXponential Enrichment) (1,2) has been a widely used *in vitro* evolution method for discovery of nucleic acid-based affinity reagents called aptamers. These reagents bind to targets such as proteins, small molecules and whole cells with high affinity (3–5), and have shown promise in a wide variety of applications, including affinity purification, histochemistry, target validation, diagnostics and therapeutics (6–9). Chemical modification of aptamers has been found to improve their performance in these applications, and the number and type of modifications continues to increase with time (10). For example, most aptamers for therapeutic applications have high molecular weight polyethylene glycol (PEG, typically 40 kDa) conjugated to the 5′ terminus to reduce renal clearance as well as 2′-ribose modifications (e.g. -CH_3_, -F, or -NH_2_) and/or phosphate modifications (e.g. phosphorothioate or dithiol) to improve nuclease stability (7,11). Other aptamer modifications include conjugation to cholesterol to increase circulating half-life (8,12) and conjugation of fluorescent dyes or gold nanoparticles for molecular imaging (13–16).

One recent development is the use of base modifications within SELEX to improve the properties of the resulting aptamers. For example, the substitution of various forms of 5-N-carboxamide-modified deoxyuridine for dT in the SELEX process has resulted in improved success rates in SELEX as well as better binding affinities for the identified sequences, some with binding constants in the low pM range (5,17). Recently developed SELEX protocols that include the use of these modified nucleotides as well as general anionic competitors have resulted in a new class of aptamers with high affinity and slow off-rates, named SOMAmers (Slow-Off Rate Modified Aptamers). The strongest binding is usually observed with variants of these modified nucleotides that contain hydrophobic aromatic moieties such as benzyl, naphthyl and tryptamino groups (5,17). These functional groups allow, e.g. interactions with protein hydrophobic surfaces that are not possible with standard DNA nucleotides (18,19), as well as allowing stacking interactions that stabilize the aptamer structural framework (20).

In view of the importance of the recent addition of these modified nucleotides, we undertook a systematic study of a portion of the current portfolio of 5-N-carboxamide modifications to determine the impact of these modifications on the thermodynamic stability of the SOMAmer reagents that contain them. Two key fundamental questions concern the impact of these modifications on double helical structures, and on the intramolecular folding of single-stranded structures.

The impact of these new modifications on canonical double helical structures is important in hybridization applications, as well providing insight into the general impact of a structural modification within double helical structures. It is also of value in the context of our multiplexed proteomic assay (SOMAscan) that utilizes hybridization of nucleic acid ligands to their complementary sequences in a microarray format (17). Toward this end, we generated two series of sequences based on two SOMAmers that bind IL-6, to understand the effect of changing the type of modification in a systematic manner.

Based on several crystal structures of SOMAmer:protein complexes, it is clear that the 5-N-carboxamide modifications also impact the intramolecular folding of single-stranded sequences. These crystal structures show that many of the hydrophobic side chains of the modified nucleotides are clustered and interact with the hydrophobic faces of the proteins (18,19). Other hydrophobic modifications stack with rings of the nucleobases and/or other modified nucleotides and appear to stabilize the internal scaffolding of SOMAmer structure (20). These features provide a partial explanation for the empirical observation that the structures of SOMAmers are far from those predicted with nucleic acid folding algorithms.

To better characterize these observations, we used spectroscopic approaches to address the impact of the base modifications on the energetic forces that drive SOMAmers to fold into their unique structures, and address whether SOMAmers structures exist in the absence of proteins to which they bind. We examined the melting of the two IL-6 SOMAmer reagents, along with a third SOMAmer that binds nerve growth factor β (NGF-β), via absorbance at 260 nm. We also examined the NGF-β SOMAmer further using circular dichroism and NMR.

As a specific example of the contribution of modifications on SOMAmer infrastructure, a zipper-like interaction is observed in the structure of the NGF-β SOMAmer (20) in which the SOMAmer structure appears to be stabilized by an interaction between two BndU nucleotides, where the uridine base of one BndU stacks with the benzyl ring of a second BndU, and *vice versa*. As a result of this particular observation, we designed a set of model sequences to determine if such stacking interactions impart measurable thermodynamic stability to a duplex.

## MATERIALS AND METHODS

### Reagents

HPLC grade acetonitrile (ACN, Sigma–Aldrich) was used in all UPLC mobile phases. All water used in these experiments was 18 MΩ water generated from an in-house system (Millipore Milli-Q Integral 10 system). UPLC vials were ‘Total Recovery’ 1 ml vials (Waters). 100 mM trieythl ammonium acetate (TEAA) was made by dilution of a commercial 2 M stock solution (Transgenomic). 100 mM tetraethyl ammonium bicarbonate (TEAB) was prepared by dilution of a 2 M TEAB stock. The 2 M TEAB was prepared by the drop wise addition of 278 ml of triethylamine to 700 ml of water while simultaneously bubbling carbon dioxide through the solution. The stock 2 M TEAB solution is stored at 4°C.

### Oligonucleotide and SOMAmer Reagent Synthesis

Oligonucleotide sequences that did not contain modified nucleotides were purchased from Integrated DNA Technologies (Coralville, Iowa). All SOMAmer reagents and other sequences containing modified nucleotides were synthesized at the 1 μmol scale via solid phase synthesis using standard phosphoramidite methods (21) with some adjustments to the protocol (e.g. phosphoramidite solvents and coupling times) to account for the base modifications. All standard phosphoramidites were purchased from Glen Research (Sterling, VA). Modified nucleoside phosphoramidite and triphosphate monomers were synthesized according to protocols described previously (18,22).

Detritylation was accomplished with 10% dichloroacetic acid in toluene for 45 s. Coupling was achieved with 0.1 M phosphoramidites in 1:1 acetonitrile:dichloromethane activated by 5-benzylmercaptotetrazole and allowed to react 3 times for 5 min. Capping and oxidation were performed according to instrument vendor recommendations. Deprotection was affected in two steps with 20% diethylamine in acetonitrile, 3 washes for 10 min each, followed by treatment with concentrated ammonium hydroxide reacted for 24 h at 37°C.

Deprotected product was evaporated to dryness in a Genevac HT-12 centrifugal evaporator, ramping the pressure down from 700 mbar to 8 mbar over 90 min followed by heated drying at 8 mbar for 15 h. After re-constitution in water, product was filtered through a 0.2 μm syringe filter and salt exchanged (from triethylammonium to sodium salt) on a 3 kDa molecular weight cut-off (MWCO) Amicon Ultracel centrifuge filter.

### SOMAmer reagent purification

Oligonucleotides obtained from Integrated DNA Technologies (Coralville, IA) were purified by the manufacturer and used without further purification. In order to obtain high purity SOMAmer reagents for thermodynamic analysis, and because the sample requirements were small, internally produced sequences were purified on an analytical scale using a Waters Acquity UPLC system, consisting of an autosampler (Model UPA), a binary pump module (Model UPB), a temperature controlled column compartment (Model UPM) and a photodiode array detector (Model UPL, extended wavelength range 190–800 nm). Purifications were done using an ion pairing reverse phase method on a Waters Xbridge C18 column (4.6 × 75 mm, 2.5 μm) at 80°C, a flow rate of 0.3 ml/min and detection at 260 nm. Each sample injection was 10 μl, and autosampler vials were maintained at 10°C. Mobile phase A was 95/5 100 mM TEAB/ACN, and mobile phase B was ACN. The column was held at 20% B for the first minute, then a gradient was applied from 20–40% B over 20 min.

During elution of the peak of interest, 12 fractions were collected of ∼30–40 μl each. The fractions were then analyzed for purity using the UPLC method described below. The purest fractions (typically 70–80%) were used in the melting experiments.

### SOMAmer purity via UPLC analysis

The purity of the SOMAmer reagent sequences was measured on a Waters Acquity system as described above. Samples were diluted ∼20X with water, and 10 μl of each diluted sample was injected for analysis. A commercial mixture of uracil, acetophenone, toluene and naphthalene (Phenomenex ALO-3045) was used as a system suitability standard for resolution and asymmetry. Analyses were done using an ion pairing reverse phase method on a Waters Acquity OST column (2.1 × 100 mm, 1.7 μM) at 80°C, a flow rate of 0.2 ml/min and detection at 260 nm. Each sample injection was 10 μl (or 1 μl for standard), and autosampler vials were maintained at 10°C. Mobile phase A was 95/5 100 mM TEAA/ACN, and mobile phase B was ACN. The column was held at 15% B for the first minute, then a gradient was applied from 15–35% B over 20 min.

### Optical melting sample preparation

Samples of SOMAmer reagents and duplexes samples were prepared in order to obtain concentrations of ∼1 μM in 140 μl of the appropriate buffer, which gave an absorbance of 0.5–1.0 AU at 260 nm. Duplex concentrations were determined by extrapolating the single stranded baseline of the duplex melt to 25°C, then using Beer's law and the sum of the two individual strand extinction coefficients. Each extinction coefficient was estimated using Equation 1, which represents a standard nearest neighbor approximation (23,24), plus an estimated correction for each modified base.

(1) \begin{equation*} \varepsilon _{260} = \sum\limits_1^{N - 1} {\varepsilon _{260NN} } - \sum\limits_2^{N - 1} {\varepsilon _{260ind} } + \sum\limits_1^{N_{mod} } {\varepsilon _{260mod} } \end{equation*}

Where: ε*_260_* = the estimated extinction coefficient of the oligonucleotide; ε*_260NN_* = the nearest neighbor coefficient for a pair of bases; ε*_260ind_* = the coefficient for an individual base; ε_260*mod*_ = the correction factor per modified base; N = the length of the oligonucleotide; N*mod* = the number of modified nucleotides.

The estimates of the ε260*mod* values are based on the changes in the intensity and the λ_max_ observed each modified nucleotide relative to dT and/or by analogy to similar structures, and were as follows ((l/molcm)*10^−3^): BndU = 6.0, FBndU = 6.0, iBudU = -0.7, IMdU = 6.0, MBndU = 6.0, MOEdU = 5.0, NapdU = 6.0, 2NapdU = 5.0, PEdU = 5.0, PyrdU = 4.4 and TrpdU = 4.3.

Each sample was heat/cooled prior to analysis to ensure proper annealing and reduce possible aggregation. Heat–cool cycles were done on a MJ Research PTC-200 Peltier Thermal Cycler, heating to 95°C and holding for 3 min, cooling to 37°C at 0.1°/s and holding at 37°C for 10 min. For high salt buffers, samples were first prepared in a smaller volume of low salt buffer for the heat/cool step, then adjusted to the final buffer conditions. Several detailed preparations are described below as examples.

For each IL-6 SOMAmer duplex in high salt buffer (1 M NaCl, 50 mM sodium phosphate, pH 7.4), 200 pmol of each strand were mixed in 70 μl of 50 mM sodium phosphate (pH = 7). Each sample was then run through a heat–cool cycle, then mixed 1:1 (70 μl/70 μl) with 50 mM sodium phosphate/2 M sodium chloride buffer.

For each IL-6 SOMAmer duplex sample in low salt buffer (130 mM NaCl, 20 mM sodium phosphate, pH 7.4), 200 pmol of each strand were mixed in 70 μl of 20 mM sodium phosphate (pH = 7). Each sample was then run through a heat–cool cycle, then mixed 1:1 (70 μl/70 μl) with 20 mM sodium phosphate/130 mM sodium chloride buffer.

### Optical melting experiments

For each SOMAmer reagent or duplex sample, 120–140 μl of the final solution was transferred to the micro-cuvette and read on a Shimadzu 1650 UV spectrophotometer at 260 nm. The cuvette holder was not moved during each individual melt, as this was found to generate baseline variability. Data were also collected while the sample was cooled at the same rate to ensure that there was no hysteresis in the transition temperature that would indicate that the heating/cooling rate was too fast. A buffer blank was subtracted from each data set. With each sample set, a natural DNA duplex with known composition and melting temperature was run as a positive control.

For the series A and series B duplexes, data were collected from 25–95°C, using a ramp rate of 0.5°C/min and a data collection rate of 0.5°C/min. For each SOMAmer reagent sample, data were collected from 5–95°C using a ramp rate of 0.5°C/min, and a data collection rate of 2 points/min. For the benzyl zipper duplexes, data were collected from 15–95°C, using a ramp rate of 0.5°C/min, and a data collection rate of 1 point/min. When samples were cooled below 25°C, a mild stream of argon gas was flowing in the sample chamber to prevent condensation.

### Optical melting T_m_ calculations

T_m_ values were calculated from A_260_ versus temperature experiments by two methods. The first was by defining and extrapolating lower and upper baselines for each sigmoid plot, which represent the double stranded (or folded) and the single stranded states, respectively. The T_m_ was then calculated as the temperature at which the A_260_ value was half way between these two baselines. Data were exported to Microsoft Excel for this analysis.

The second method was by determining the maximum of the first derivative of the absorbance signal (dA_260_/dT) from the melting curve. This value was calculated by exporting the data to Microsoft Excel, and calculating a 5 or a 9 point slope as a function of temperature, and manually determining the maximum. It should be noted that for duplexes of non-identical strands, this value differs from the true T_m_ by 1–2°C. (See "Results and Discussion, Comparison of Tms within the series A and series B".) For series A and series B, each T_m_ value reported via this second method was an average of two or more independent experiments. Differences between the two runs ranged from 0.0–1.2°C, with one exception, which was 3.4°C. (See text.) Melting temperatures for intramolecular transitions of SOMAmer reagents were determined using this method.

### Determining thermodynamic parameters

Thermodynamic values for the IL-6 duplexes were obtained using two methods which require the approximation of a two state model: a standard van't Hoff approach (25–27), and from the slope method ΔH° = 6RT_m_^2^(dθ/dT)_T = Tm_ (27,28). The van't Hoff analysis was limited to the range 0.15 < θ < 0.50 to reduce the contribution of impurities with lower T_m_s, as well as limit the contribution of folded states of the single strand upper baseline, both of which are expected to reduce errors associated with a two-state approximation. Details of the methods for estimating thermodynamic parameters can be found in the Supplementary Information. While the two-state approximation certainly influences the accuracy of the ΔH° and ΔS° values obtained by these methods, the systematic nature of the errors within each series of duplexes, the agreement between the two methods, and the linearity of the van't Hoff plots allow meaningful determination of the impact of the modifications.

### Circular dichroism

Circular dichroism spectra were collected using a Jasco Model J-810 spectropolarimeter. Spectra were collected in the range 5–85°C, at intervals of 10°C, in the 320–200 nm range, at a resolution of 0.5 nm, a scanning rate of 50 nm/min, a bandwidth of 1 nm, a response of 2 s, 6 accumulations per scan, at standard sensitivity (100 mdeg). The SOMAmer reagent concentration was 1 mg/ml (0.1 mM) in 130 mM NaCl, 20 mM Na-phosphate, pH 7. The cell pathlength was 1 cm.

### NMR

The NGF SOMAmer (OH-2426–66_50) was prepared as a 1 mM SOMAmer solution in 130 mM NaCl, 20 mM sodium phosphate, 1 mM EDTA, pH 7. It was then lyophilized and re-dissolved in (H_2_O/D_2_O 90/10). 300 μl was placed in a Shegemi 5 mM tube, susceptibility matched for the H2O/D2O solvent. The NMR spectra were acquired using a Varian Inova 600 NMR spectrometer operating at 599.71 MHz for the ^1^H observe channel equipped with room temperature z-axis pulsed-field gradient probe. Suppression of the solvent resonance was accomplished using a gradient optimized 1:1 spin-echo pulse sequence. Other pertinent acquisition parameters include: ^1^H 90-degree pulse of 7.5 μs, 128 scans, relaxation delay of 2.5 s, an acquisition time of 1.36 s, and a spectral width of 15 015 Hz. Sample temperatures were corrected versus displayed temperatures using standard methods with ethylene glycol using identical air-flow conditions as with the analytical sample.

The two strands of the benzyl zipper duplex were mixed in a 1:1 ratio to achieve a final duplex concentration of 1 mM in 130 mM NaCl, 20 mM sodium phosphate, 1 mM EDTA, pH 7. It was then lyophilized and re-dissolved in 99.9% D_2_O (Cambridge Isotope Laboratories).

Pulsed field gradient optimized DQF-COSY, TOCSY and NOESY data were collected using a Varian VNMRS-800 NMR spectrometer operating at 799.21 MHz for the ^1^H observe channel, with a ^1^H 90-degree pulse of 11.75 μs duration. All 2D NMR spectra were acquired as phase-sensitive data sets using the States–TPPI method for pure-phase and quadrature in the t1 dimension. All 2D spectra were acquired using a spectral width of 7225 Hz (9.0 PPM) in both dimensions. Zero-filling was applied for all spectra in t_2_ dimension and Linear Prediction was applied in the t1 dimension, yielding a 2K x 2K matrix after Fourier transformation. DQF COSY data were collected using 1024 complex points in t2 for each of 1024 t_1_ values, with 16 scans per t_1_ increment. TOCSY data were collected using 1024 complex points for each of 512 t_1_ values, with 8 scans per t_1_ increment. Spectra using mixing times of 20 ms and 80 ms were acquired under a MLEV-17 spin-lock sequence to identify short-range and long-range through-bond correlations. The NOESY data were collected using 1024 complex points for each of 512 t_1_ values, with 16 scans per t_1_ value. NOE Mixing times of 120 ms, 150 ms and 250 ms were measured to ensure that cross peak intensities were still increasing monotonically versus mixing time for relative distance determination.

## RESULTS AND DISCUSSION

### Design of duplex sequences containing modified nucleotides

The 5-(N-carboxamide)-2′-deoxyuridine nucleotides that form the core of these studies are shown in Table 1. Two series of sequences were created based on substitutions within the sequences of two SOMAmer reagents, which are shown in Table 2. The two ‘parent’ SOMAmer sequences were selected and optimized for binding to IL-6, a pleiotropic cytokine with an important role in immune regulation, hematopoiesis, inflammation and oncogenesis. The biochemical properties of these SOMAmer reagents (27) and a crystal structure (19) have been recently published. The first sequence, SL1023, is a 32-mer that contains 10 BndU nucleotides (K_d_ ∼1 nM), though the 8 BndU and 7 BndU/1 NapdU variants are also active (K_d_ ∼2 nM). The crystal structure was obtained for a variant containing eight BndU nucleotides, a NapdU at position 12, a PEdU at position 9 and 6 2′-OCH_3_ substitutions (K_d_ ∼0.2 nM). The second sequence, SL1030, contains 39 nucleotides, seven of which are NapdU (K_d_ ∼0.2 nM). This was later shortened to an active form containing 28 nucleotides and 6 of the original NapdU nucleotides with no loss in activity (29).

**Table 1.**
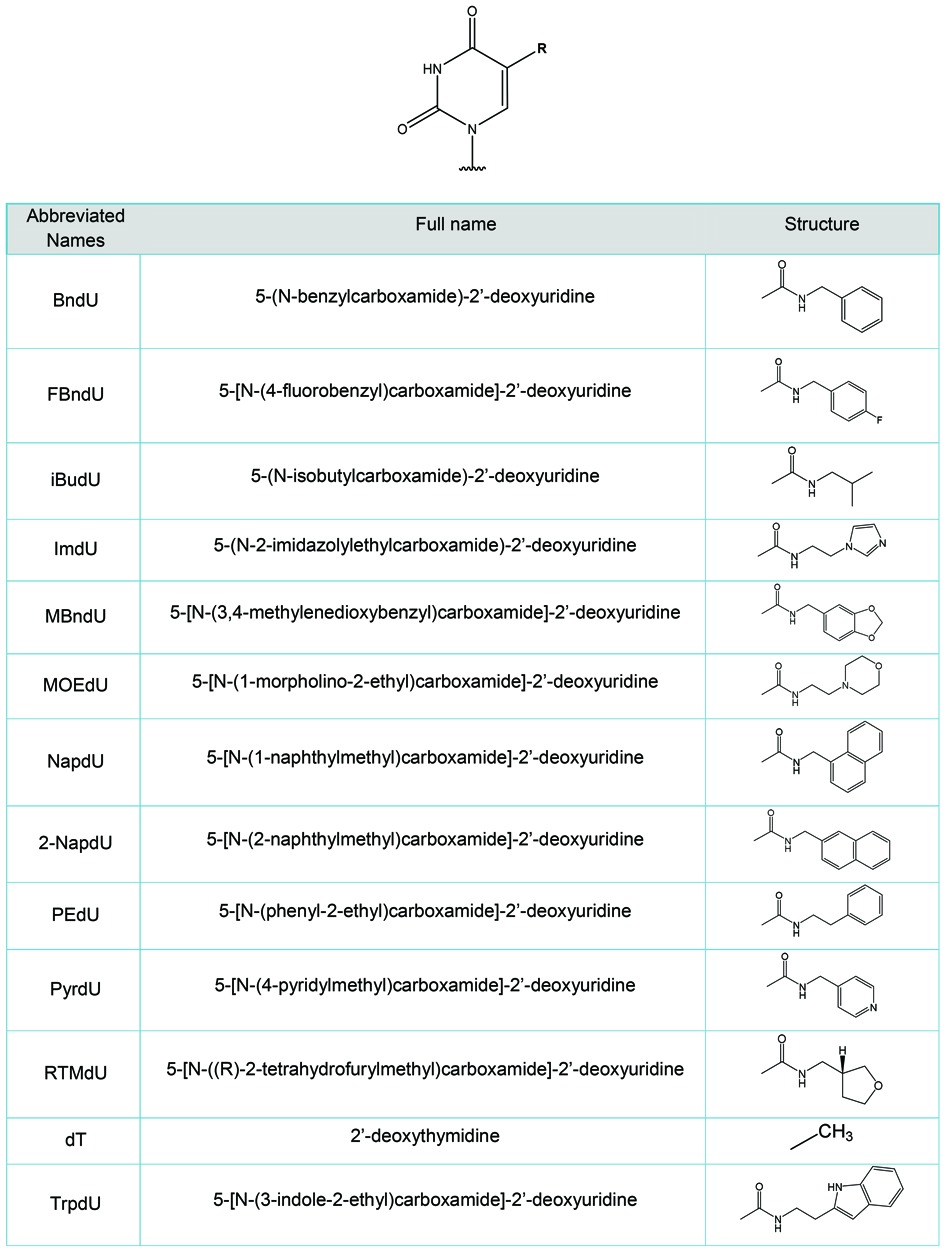
5-N-carboxamide Nucleotides (sugar and phosphate not shown)

**Table 2.**
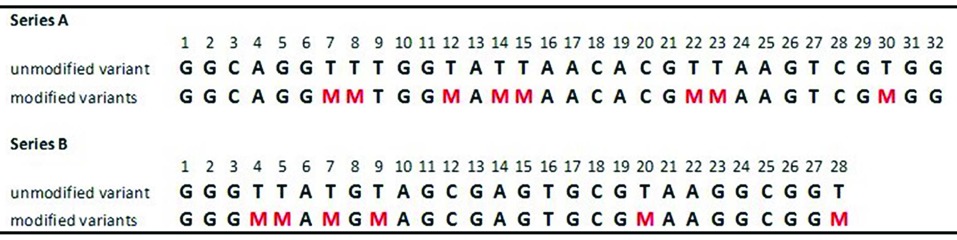
Sequences studied to understand the impact of modified nucleotides on duplex stability. In Series A, variants include all 8 positions substituted with M = BndU (e.g. 8-BndU), FBndU, iBdU, ImdU, MBndU, MOEdU, NapdU, 2NapdU, PEdU, PyrdU, RTMdU, ThrdU, or TrpdU and a BndU variant with 1 NapdU at position 12 (7BndU/1NapdU). In Series B, variants include all 6 positions substituted with the same 5-carboxamide modified nucleotides with the exception of RTMdU and mixed BndU/NapdU variant

The two series of sequences were designed to examine the effect of changing the type of modification in a systematic way. Series A is based on the 8 BndU variant of SL1023, in which all eight modified nucleotides were systematically substituted with eight identical modifications of another type. Series B is based on the 6 NapdU variant of SL1030, with a similar set of uniformly substituted analogs. Because this sequence has a modified nucleotide at the 3′-terminus, and modified nucleotides are not available as CPG reagents, a dC was added to the 3′ terminus of each sequence. A corresponding dG was added to the 5′-terminus of the complementary sequence, allowing formation of a duplex of length 29 nucleotides. The RTHF variant was not included in this series, nor was an analog of the 7 BndU/1 NapdU variant.

### Comparison of T_m_s within the series A and series B

We first examined the impact of the 5-N-carboxamide modifications on the stability of fully based paired DNA duplexes. Toward this goal, we analyzed each of the sequences in Series A and Series B in duplex form with its natural DNA complementary sequence, which is the same for all of the variants in each series. Representative melting curves are shown in Supplementary Figure S1. These curves for the 8-NapdU, natural DNA and 8-iBudU variants in Series A, and the 6-NapdU, natural DNA and 6-iBudU variants in Series B show that these modifications have a significant impact on the duplex stability. For example, in Series A, the T_m_ value of 8-NapdU duplex is destabilized by 7°C relative to the natural DNA duplex under high salt conditions (1 M NaCl), and the 8-iBudU duplex is stabilized by 4°C. It is also interesting to note that total hypochromicity for the each modified duplexes is similar (19% and 20%, respectively), and lower than that of the all-DNA duplex (24%). The same pattern for the relative effect of these three modifications is observed for series B, though the magnitude of the T_m_ changes is smaller. The reduced hypochromicity observed with the formation of the modified duplexes may reflect reduced stacking of the bases in the double stranded structure, caused by the perturbation of the modifications situated in the major groove. Conversely, because the value represents a percent change, it could also reflect stronger base stacking present in single stranded structures due to the presence of the modifications combined with little or no perturbation in the duplexes.

The T_m_ values for the duplexes formed by each of the sequences from the two series are summarized in Table 3, ranked from lowest to highest. Each T_m_ measurement reflects the maximum of the first derivative of the absorbance signal (dA_260_/dT). For a subset of the melting curves, the T_m_ values were also determined as the temperature at which θ = 0.5, where θ is the fraction of strands in the double stranded state, and represents the true definition of the T_m_ for a non-self-complimentary duplex. As predicted (26), the T_m_ values determined via inflection point are higher than the true T_m_ values by 0.2–1.0°C. Comparisons of the sequences within each series were made using the derivative method.

**Table 3.**
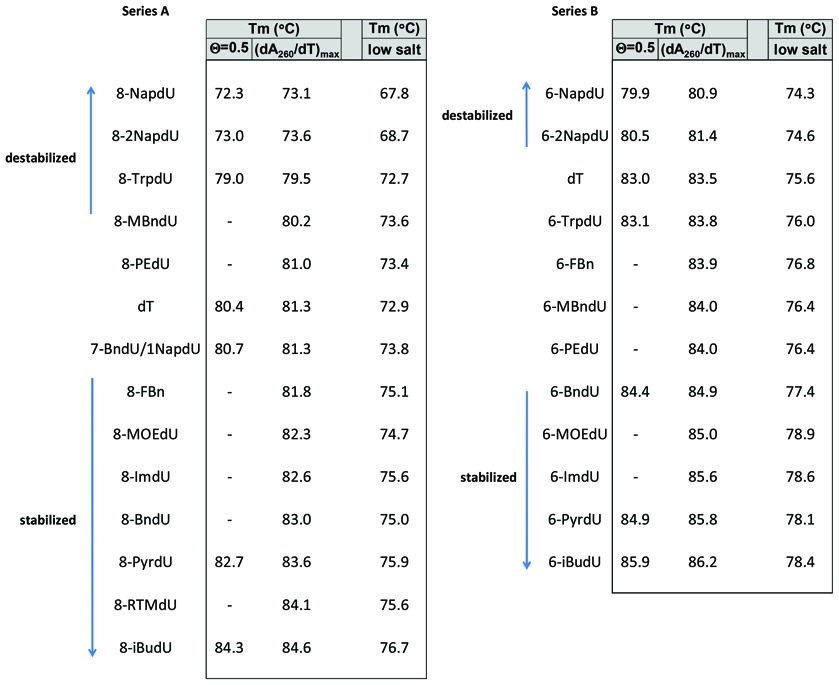
Melting temperatures of oligonucleotide duplexes from series A and series B (based on A_260_ versus temperature). T_m_ values were measured as (dA_260_/dT)_max_ under both high salt (1 M NaCl, 50 mM sodium phosphate, pH 7.4) and low salt (130 M NaCl, 20 mM sodium phosphate, pH 7.4) conditions. For comparison, T_m_ values were calculated as θ = 0.5 for a subset of duplexes in each series

Of the modifications tested, the most pronounced destabilization relative the all-DNA duplex was observed with the two-ring hydrophobic aromatic modifications (NapdU and 2NapdU) in both series (ΔT_m_ values in the range of −2.1 to −8.2°C). The more polar two-ring modifications, TrpdU and MBndU, are modestly destabilizing in Series A and neutral in Series B.

The polar modifications (IMdU, MOEdU, PyrdU and RTHFdU) stabilized the double helix relative to unmodified DNA (ΔTm values in the range of 1.0 to 2.5°C). The smaller non-polar aromatic groups, including BndU, FBndU and PEdU, had an effect ranging from neutral to modestly stabilizing (ΔT_m_ values of 0.3 to 1.7°C). The relatively small aliphatic iBudU modification was the most stabilizing modification of those tested ((ΔT_m_ value of 2.7 to 3.3°C).

The differences between the low-salt and high-salt T_m_ values are consistent with recent theory, which predicts that T_m_ values have a quadratic dependence on sodium concentration (30). The general pattern observed between the high- and low-salt conditions is similar, but there does appear to be a differential effect on the hydrophilic modifications (Supplementary Table S1). For example, in the high-salt buffer, for the average change in T_m_ from the unmodified duplex for MOEdU, ImiddU and PyrdU is 2.0°C. This average value increases 2.5°C in the low-salt buffer. This is consistent with expectations, since the forces that allow polar groups to show greater stability than hydrophobic groups are likely to be electrostatic in nature, and could therefore be weakened by higher counterion concentrations.

### Thermodynamic analysis for series A and series B

To better understand the impact that the 5-N-carboxamide modifications have on thermodynamic stability of DNA duplexes, it is of interest to understand whether the observed changes are driven by enthalpy, entropy, or a combination of the two. Determination of thermodynamic values from optical melting curves of DNA duplexes is based on the two-state model assumption (uniform duplexes and uniform single strands), which is certainly approximate for molecules of this size and complexity. The impact of additional states on the extracted thermodynamic parameters clearly depends on the both their abundance and the magnitude of the differences in energy from the expected dominant states of the duplex and single strands. (See Supplementary information.)

Within these two series of sequences, it is highly likely that the deviations from the two-state model will be systematic. Therefore, although the values of the measurements are likely to have error, the trends in changes observed between the duplexes are almost certainly valid. Within this approximate treatment (Table 4), the following trends were observed. For the bulky hydrophobic aromatic modifications NapdU and 2NapdU, the ΔΔH° was strongly destabilizing relative to the standard DNA duplexes, while Δ(TΔS°) was stabilizing. For sequences containing iBudU, the ΔΔH° was strongly stabilizing relative to the unmodified duplex, and Δ(TΔS°) contribution was destabilizing. The more hydrophilic modifications such as PyrdU, ImiddU and MOEdU were stabilizing relative to the unmodified duplex, again dominated by the enthalpic term. In all cases within Series A and Series B, changes in stabilization relative to unmodified duplex are the result of offsetting effects of enthalpy and entropy, with the enthalpic effect being dominant.

**Table 4.**
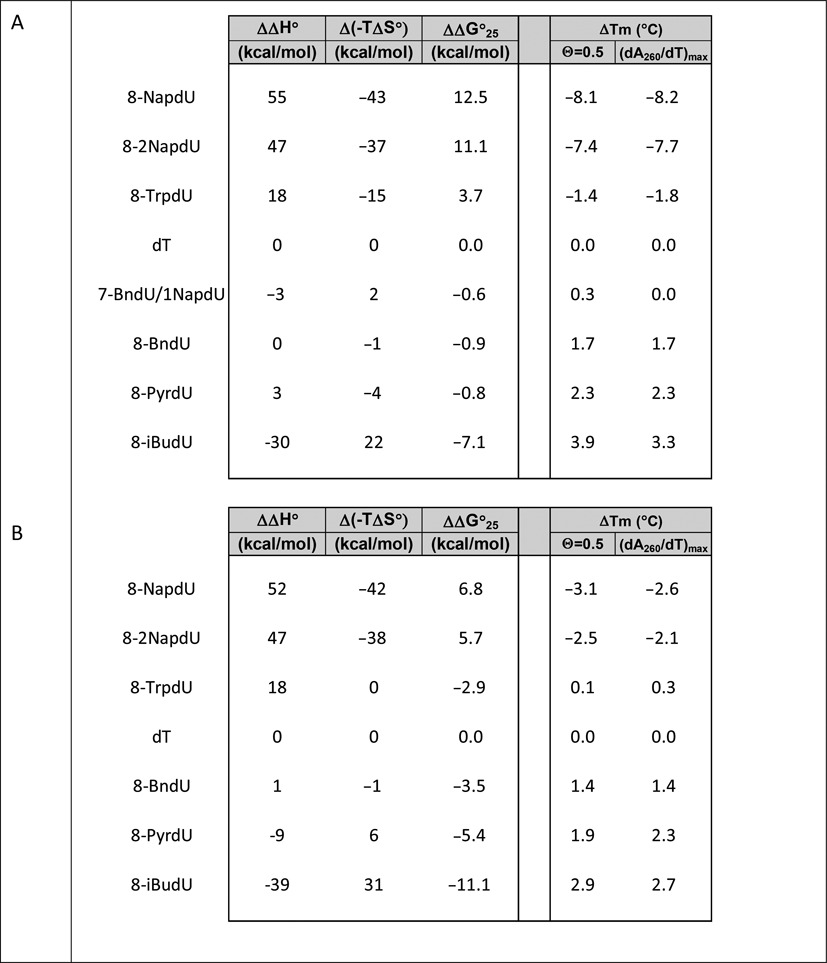
(A) Approximate changes in thermodynamic parameters for formation of oligonucleotide duplexes from Series A and B, relative to the standard DNA control, under the assumption of a two state model. Parameters were obtained via from van't Hoff analyses of optical melting at 260 nm in high salt (1 M NaCl, 50 mM sodium phosphate, pH 7.4)

The offsetting effects of DNA modifications have been observed previously within duplexes containing mismatches (25), 2′-fluoro-nucleotides (31) and locked nucleic acids (LNAs; 32). McTigue *et al*. (32) did a detailed analysis of the errors in the measurements to ensure that the results were not an artifact of the van't Hoff analysis. The physical basis of these opposing effects is generally attributed to the enthalpic gain of improved stacking, which is offset by the associated loss in degrees of freedom.

It should be noted the changes in thermodynamic stability caused by the 5-carboxamide modifications can be due to changes that affect the single-stranded states, the double-stranded states, or a combination of the two. For the bulky hydrophobic aromatic modifications NapdU and 2NapdU, one possible explanation is improved stacking of the aromatic rings in the single-stranded state, which would enthalpically stabilize the single-stranded state of the modified strand relative to analogous natural DNA single strand. If the two double-stranded states were similar enthalpically, the overall effect would be a reduction of the ΔΔH° value for the modified duplex. With respect to entropy, such a folded single-stranded state would be more ordered, and therefore would increase the Δ(TΔS°) term, as is observed. In order for this explanation to be correct, these energetic terms would have to be dominant over other contributions, such as the possible stabilizing effect of the hydrophobic groups on the stacking interactions in the double-stranded state, as well as the relative contribution of solvent water on both states. The physical explanation for the stabilization caused by the iBudU and hydrophilic modifications observations is less clear.

### The impact of 5-(N-Carboxamide) modifications on SOMAmer folding

We next examined the impact of the 5-N-carboxamide modifications on intramolecular folding of single stranded structures. Outside of the constraints of the canonical double helix, which is presumably dominant when complementary strand are present, additional novel hydrophobic interactions become possible. For example, recent crystallographic studies of SOMAmer:protein complexes show clearly that hydrophobic aromatic side chains form stacking interactions that stabilize the internal structural framework of SOMAmers (18–20). In addition, many of the side chains interact with complementary hydrophobic surfaces of the protein (18,19).

To assess the contribution of modified nucleotides on the thermodynamic stability of folded SOMAmers, we used spectroscopic methods to compare SOMAmer sequences with their natural DNA analogs. The strength of this approach is that is it very easy to compare two sequences using these methods. The key weakness is that there is no way to ensure that the natural DNA analog will fold in the same fashion, which would be required to claim the differences observed can be quantitatively attributed to the presence of the modifications. Nonetheless, an increase in stability observed in the sequence with modifications would suggest that the modifications facilitate the formation of structures that are energetically favorable over *any* structure that can be formed by the natural sequence, providing evidence that these modifications stabilize folded SOMAmer structures.

### Intramolecular melting of three SOMAmers via absorbance versus temperature

**Figure 1.**
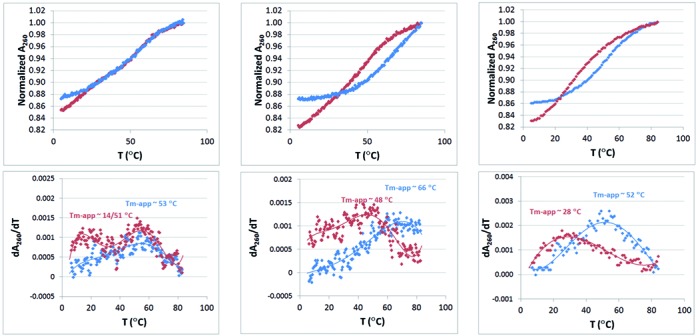
Optical melting curves (A_260_ versus temperature) for two IL-6 SOMAmers (the 7BndU/1NapdU variant from Series A and the 6-NapdU variant from Series B) and an NGF-β SOMAmer (SL1047) compared to their all DNA analogs in 130 mM NaCl, 20 mM Na-phos, 1 mM EDTA, pH 7 and the corresponding derivative plots. Plots in blue represent to the SOMAmers, and the plots in red present the corresponding all DNA analogs.

Figure 1 shows the optical melting curves (A_260_ versus temperature) for two of the IL-6 SOMAmers (the 7 BndU/1 NapdU variant of Series A and the 6-NapdU variant of Series B) and one NGF SOMAmer, compared to their natural DNA analogs. The sequences for these molecules are shown in Table 5. T_m_ values were determined from the maximum of the first derivative plot (dA_260_/dT). Each SOMAmer sequence shows sigmoidal behavior, except the 6-NapdU variant of Series B (Figure 1, center), which melts at a high enough temperature that no upper baseline is observed. These sigmoidal curves indicate transitions from structured states to less structured states. In two of the three cases shown in Figure 1, the Tm values show that sequences containing the base modifications show a significantly higher apparent Tm than their unmodified analogs (ΔT_m_s of 16 and 24°C), indicating that the modifications provide increased thermodynamic stability. The same was observed with two PDGF-B SOMAmers that we described recently (ΔT_m_s of 34 and 36°C; 18). The only possible exception to this strong trend was the 7BndU/1Nap variant of Series A (Figure 1, left panel). This SOMAmer showed a Tm of 63°C, while the unmodified DNA analog showed a biphasic transition with maxima at 59 and 21°C. Because the physical basis of the biphasic transition is not understood (e.g. independent melting of more than one conformers versus segmental melting of two regions), a direct comparison of the apparent T_m_s is complicated.

**Table 5.**
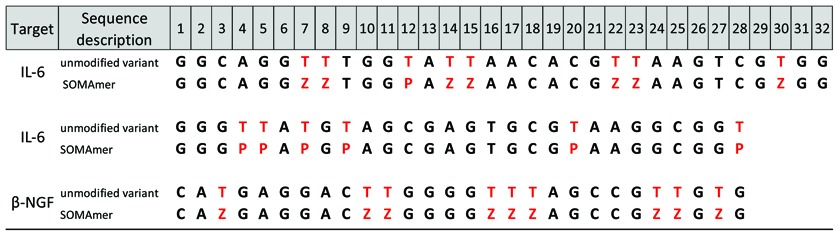
Sequence of three SOMAmer sequences and their all DNA analogs (Z = BndU, P = NapdU)

The hypochromicity of the unmodifed analog was somewhat larger than the corresponding SOMAmer sequence in each case (15–22% versus 13–14%, respectively), suggesting that more base stacking is present in these folded structures relative to the SOMAmers. This is consistent with the presence of less Watson–Crick base pairing in the SOMAmer structures, where some of the thermodynamic stabilization can be driven by hydrophobic stacking interactions involving the 5-carboxamide groups. For example, in the crystal structure of the NGF SOMAmer, no Watson–Crick base pairs are observed (20). These interactions may not generate the same level of hypochromicity observed with the stacking of nucleobases in standard DNA geometries. Taken together, these results show that modified nucleotides provide thermodynamic stability not possible with unmodified nucleobases. It is also clear that the presence of the protein is not required for the SOMAmers to maintain folded structures at lower temperatures. It is interesting to note that NapdU was destabilizing in the context of a duplex structure, but is stabilizing within an intramolecular structure, supporting the notion that intramolecular folding allows formation unique hydrophobic interactions.

### Comparing the intramolecular melting of an NGF-β SOMAmer via absorbance, circular dichroism and NMR

To ensure that the transition observed in the optical melting experiment corresponds to a global unfolding event, the melt for the NGF SOMAmer was repeated using circular dichroism (CD) to observe the transition. CD, which is the differential absorption of left and right circularly polarized light, is sensitive to conformational changes in biological macromolecules. In DNA, the signals result mainly from changes in the dipole–dipole interactions between nucleobases, and the technique can be used, e.g. to distinguish A, B and Z forms of DNA, as well as helix-to-coil transitions (33).

The CD spectrum of SL1047 in 130 mM NaCl, 50 mM sodium phosphate, pH 7 (analogous to the optical melting data reported in 20) is shown in Supplementary Figure S4A. A strong change is observed in the signal intensity and peak maximum in the positive band centered near 280 nm as a function of temperature. A strong positive band in the 260–280 nm range is typical of B-form DNA (33) although this SOMAmer is not expected to have significant helical structure based on the co-crystal structure (20). A plot of the CD intensity at 280 nm as a function of temperature is shown in Supplementary Figure S4B, with an apparent T_m_ of ∼48°C, in good agreement with the T_m_ value observed via melting at A_260_ under these conditions (52°C), strongly indicating that the same transition is observed by both techniques. An isosbestic point is observed at 256 nm, consistent with the presence of a two-state transition (34).

As a third evaluation of this transition, the imino proton region of the ^1^H NMR spectrum of SL1047 was examined as a function of temperature. Typically, the imino protons of nucleic acid bases exchange very quickly with bulk water protons and are therefore not observed in standard one-dimensional ^1^H NMR spectra. When these protons are involved in hydrogen bonding interactions in standard Watson–Crick or other stable structures such as Hoogsteen base pairs, however, the exchange rate is sufficiently slow that they can be observed (35,36). Similarly, these protons may be observed if they are involved in a structural moiety that has limited solvent accessibility such as hairpin loops or hydrophobic pockets.

Guanosine-N^1^ imino protons in a GC base pair are typically observed in the 12–13 ppm region, and thymidine-N^3^ imino protons in an AT base pair are usually observed in the 13–14 ppm region (37). Imino protons observed in the 10–12 ppm region have been assigned to structures such Ts in loops or base triplets (38), G-U and G-A base pairs (39) and G-quartets (40).

**Figure 2.**
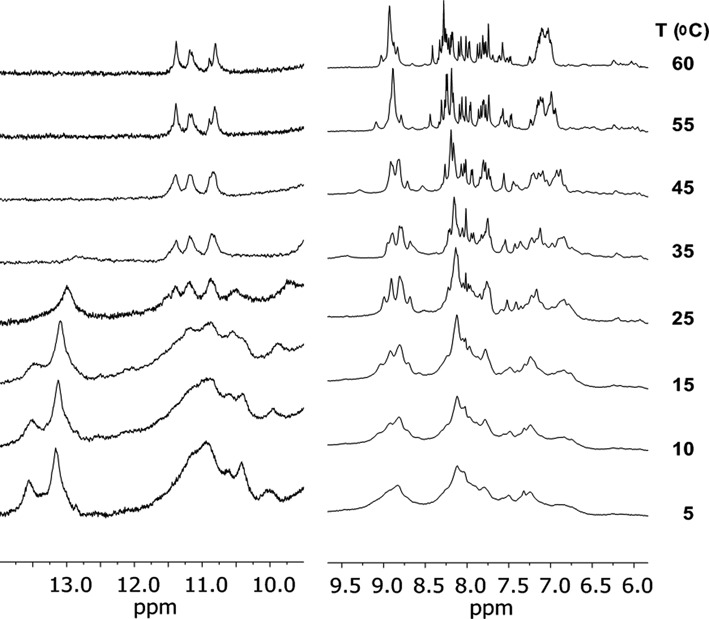
Imino and Amino regions of the proton NMR spectrum (at 600 MHz) of the β-NGF SOMAmer SL1047 (1 mM SOMAmer, 130 mM NaCl, 20 mM Na-phos, 1 mM EDTA, pH 7 (H_2_O/D_2_O 90/10)) using a 1:1 spin-echo. The scale of imino region has been expanded to 6X relative to the amino region to facilitate visualization.

Figure 2 shows the imino and amino regions of the ^1^H NMR spectrum OH-2426–66_50 using a gradient optimized 1:1 spin-echo to suppress the water (HDO) resonance. At the lowest temperatures, a variety of broad imino resonances are observed in the 10–12 and 13–14 ppm region. The large number of imino proton resonances observed shows that the SOMAmer has some degree of defined structure in solution at low temperature, independent of the target protein. The broad line widths observed, however, suggest that a number of structures could be in exchange on the NMR time scale.

As the temperature is raised, the loss of resonances show that the structure is becoming more dynamic, allowing some exchange of the imino protons with bulk solvent. By 37°C, a significant fraction of the resonances are gone, indicating that under physiological conditions, the structure is either partially unfolded or in a dynamic equilibrium between folded and unfolded structures. It is interesting to note that a portion of the imino resonances (10.5–11.5 ppm) persist up to 60°C, showing that a portion of structure is exceptionally stable.

One of the most interesting observations is that the imino resonances are not disappearing in a uniform fashion as a function of temperature, showing clearly that the transition is not two-state, which is intuitively reasonable. Short natural DNA double helices are thought to ‘unzip’ in a highly cooperative process over a narrow temperature range (26). In contrast, many SOMAmers (and aptamers as well) are likely to be an aggregate of partially isolated structural entities. In the crystal structure of the OH-2426–66_50, e.g. several different regions are observed, including a benzyl zipper and a hydrophobic pocket (20). It is reasonable that these regions could unfold at independent temperatures rather than in a cooperative process. Consequently, use of thermodynamic calculations that require the assumption of a two-state model for folding of SOMAmers would be approximate at best, and the midpoint of the transition observed by the optical methods is probably more correctly characterized as an ‘apparent T_m_’. Thus, what appeared to be a two-state transition based on the CD spectra, is revealed to be more complex by the NMR data.

Figure 2 also shows the amino proton region of the ^1^H NMR spectrum (∼7–9 ppm), which overlaps with the aromatic proton region. The disappearance of these resonances, also involved in hydrogen bonding in canonical base pairs and other structures, are observed to disappear in a pattern that is consistent with what is observed in the imino region of the spectrum.

### Thermodynamic contribution of a benzyl zipper motif

**Figure 3.**
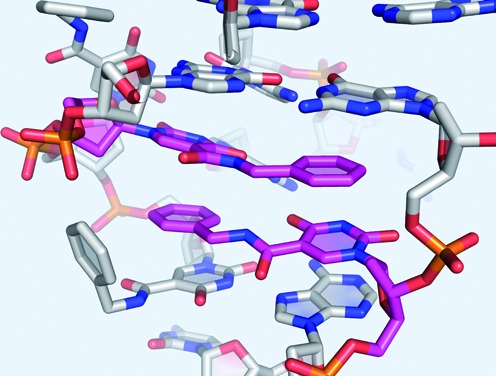
Benzyl zipper structure observed in the crystal structure of SL1047 in complex with β-NGF (20), showing the zipper-like interaction of BndU_3_ and BndU_16_.

The crystal structure of SL1047 with NGF-β shows a unique and interesting structural motif involving two BndU nucleotides from different parts of the chain interacting in a fashion that presumably provides structural integrity to the folded SOMAmer (20). In this motif (Figure 3), the uridine ring of U_3_ stacks with the benzyl ring of U_16_ and *vice versa* in a zipper-like interaction. Stacking interactions among non-natural nucleotides have been observed previously in the context of new base pair candidates that cannot form canonical hydrogen bonds (41,42). The 5-carboxamide groups are different, however, in that these modifications allow canonical hydrogen bonding to be maintained.

**Figure 4.**
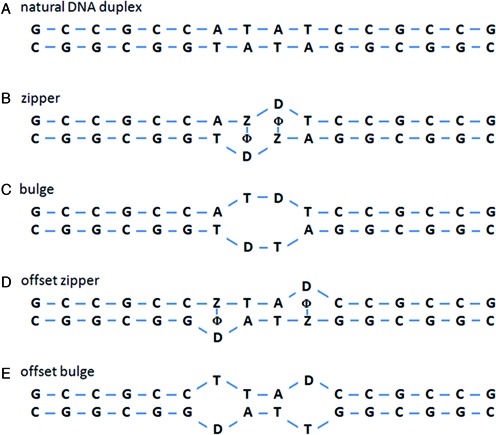
Model sequences designed to measure the thermodynamic contribution of the benzyl zipper motif. In these sequences, Z = BndU, with ϕ representing the benzyl ring of this nucleotide, and D = d spacer, which is an abasic nucleotide designed to provide space to accommodate the benzyl rings.

It is of interest to quantitate the contribution of this structure to thermodynamic stability, although it is inherently difficult to do. As a first step, one can, in principle, create an analog sequence without the key modifications to compare to the SOMAmer in a thermodynamic analysis, but there is a risk, as mentioned above, that this analog sequence will fold into a completely different core structure. In an attempt to avoid this problem, we designed a series of model duplex sequences in an attempt to ensure the formation of zipper structure and minimize the risk that alternate folding structures could confound the interpretation of the data. These model compounds are shown in Figure 4.

Figure 4A shows the control duplex, a canonical double stranded sequence with CG stems anchoring a central AT core. In the zipper model (Figure 4B), the two central dT nucleotides, which are on opposite strands, are replaced with BndU nucleotides. In addition, the dA nucleotides which oppose these positions are replaced with abasic nucleotides (labeled ‘D’), with the expectation that the missing base in these nucleotides would create the space needed for the benzyl rings (represented by ϕ in Figure 4B and D) to form the zipper motif. As a control, we also designed a model duplex with thymidines replacing the BndUs (‘bulge’, Figure 4C). The lack of bases creates a central bulge in the duplex, which is expected to be less stable than the fully base-paired sequence. If the modified nucleotides create significant thermodynamic stability through stacking interactions, then the zipper sequence should be more stable than the bulge sequence. To ensure that any observed stability gain was the result of adjacent D-Z pairs, as opposed to individual D-Z pairs, we designed a second set of controls with the same two D-Z pairs in positions that are not adjacent (the ‘offset zipper’, Figure 4D, and the ‘offset bulge’, Figure 4E).

**Figure 5.**
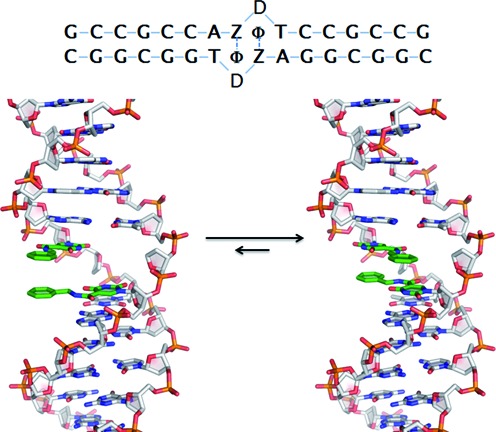
Idealized structures for the benzyl zipper duplex. On the left is true B-from structure, with the BndU nucleotides in an *anti* conformation, resulting in the benzyl groups stacked in the major groove. On the right, the glycosidic bonds of the BndU have been rotated into the *syn* position, allowing formation of the zipper motif, and the helix has been rotated to better visualize the stacking of the rings. The top view of the zipper is shown upper right.

One potential flaw in this design is that within a B-form DNA helix, modifications that originate from the 5-position of a dU base point toward the major groove, and not toward the center of the helix. Simple modeling of the zipper sequence in Figure 5 with B-form constraints show that, coincidentally, the two benzyl groups are able to stack in the major groove. Formation of the proposed benzyl zipper motif inside the helix is possible, but requires rotation of glycosidic bonds of the BndU nucleotides into a *syn* conformation (Figure 5, right). The *syn* conformation is common for purine nucleotides, and is observed, e.g. for dG nucleotides in Z-DNA (43). It is less common for pyrimidines, presumably due to steric hindrance of the C^2^-carbonyl, but has been observed (44,45). The stacking of the pi systems of the two pairs of ring structures is similar between crystal structure (Figure 3) and the model compound with BndU nucleotides in the *syn* conformation. One difference is that the amide proton and the amide carbonyl in the side chain of each of the stacked modified nucleotides in the crystal structure are in a *trans* conformation, while in the model compound these groups are *cis*. The contribution of this difference to enthalpic stabilization of the duplex is expected to be small compared to the dipole-induced dipole interaction of aromatic rings.

**Figure 6.**
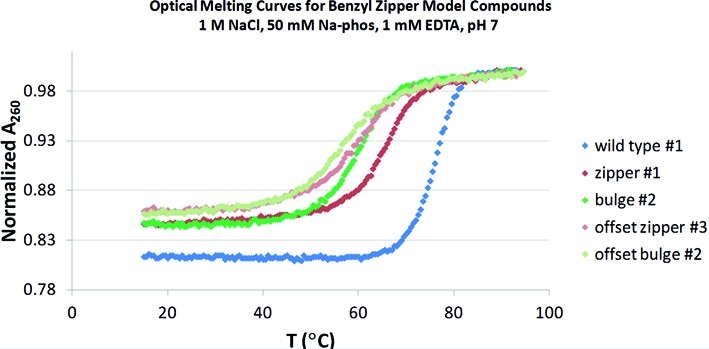
Optical melting curves (A_260_) for the model sequences designed to measure the thermodynamic contribution of the benzyl zipper motif. (See Figure 5.)

Figure 6 shows the optical melting curves for the 5 model duplexes, normalized to an absorbance of 1.0 at 95°C. The unmodified duplex has a T_m_ of 76°C, and shows the sharp transition and hypochromicity (20%) characteristic of a Watson–Crick double helix of 16 base pairs. The zipper motif shows a T_m_ value that is significantly higher than the bulge control (65°C versus 59°C, respectively), suggesting that the benzyl modifications are imparting some thermodynamic stability to the model duplex, but not as strong as the natural DNA structure. The offset zipper duplex has a T_m_ of 59°C, confirming that the stability of zipper structure is related to the nearest neighbor arrangement of the BndU nucleotides. In other words, duplexes containing two adjacent non Watson–Crick pairs (the benzyl zipper and the bulge) are less destabilizing than the analogous structures containing a pair of separated mismatches (the offset zipper and the offset bulge, respectively). In addition, the change in the% hypochromicity is also larger for the offset zipper and the offset bulge. The difference in the bulge and the offset bulge is consistent with the change in the number of nearest neighbor interactions (12 for the bulge, 11 for the offset bulge, versus 15 for the natural DNA duplex). The percent hypochromicity of the zipper is comparable to the bulge, so the base stacking in the zipper does not appear to be contributing significantly to the optical changes.

Table 6 shows the thermodynamic values for each of the model duplexes, based on a van't Hoff analysis (lnK versus 1/T within each curve, using 0.15 < θ < 0.50), with an average of three independent runs. These data follow the same patterns observed with the melting temperatures, where the zipper duplex has ΔH°, ΔS° and ΔG_25_° values intermediate between the natural DNA and the bulge duplexes. Optical melting studies indicate the zipper structure yields an enthalpic contribution of 19 kcal/mol relative to the bulge control duplex, and stabilization of the ΔG_25_° of 3.4 kcal/mol. Although the enthalpic contribution of the zipper is not as strong as the natural DNA sequence upon duplex formation, the entropic term is less unfavorable, despite the adoption of the *syn* conformations of the modified nucleotides.

**Table 6.**
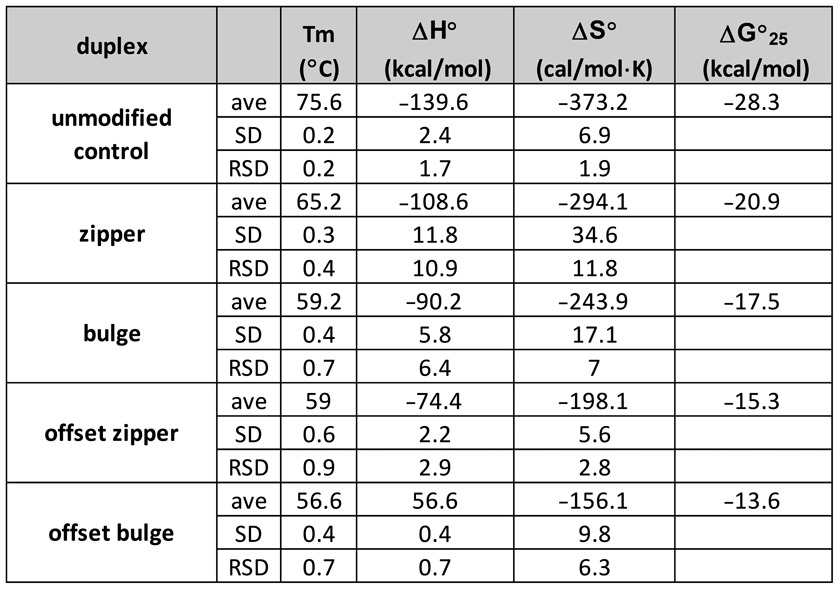
Summary of thermodynamics derived from optical melt analysis of the model sequences designed to measure the thermodynamic contribution of the benzyl zipper motif (benzyl zipper duplex, bulge control and natural DNA control)

Because the benzyl zipper model compound showed improved stability relative to the control compound containing the bulge, it is clear that some structural feature of the modified nucleotides is imparting thermodynamic stability. In order to determine if this gain in stability was due to the proposed zipper motif, two-dimensional NOESY (Nuclear Overhauser Effect SpectroscopY) experiments were conducted on the benzyl zipper duplex (0.7 mM duplex in 130 mM NaCl, 20 mM Na-Phos, 1 mM EDTA, pH 7).

**Figure 7.**
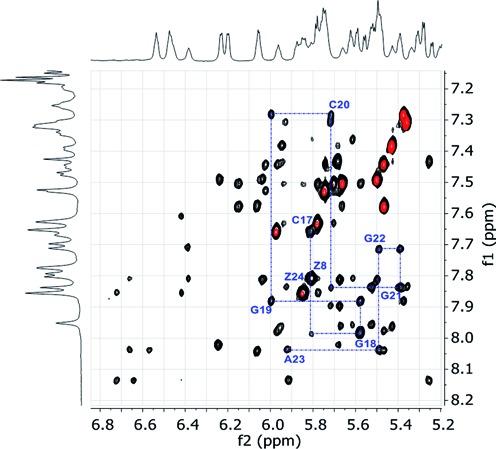
Overlap of aromatic/H1’ regions of the NOESY spectrum (black cross-peaks) and TOCSY spectrum (red cross-peaks) of the proposed benzyl zipper duplex in (0.7 mM duplex in 130 mM NaCl, 20 mM Na-Phos, 1 mM EDTA, pH 7, 40°C). The blue line traces the assignments of C_17_ to A_23_.

NOESY spectra were used to assign the base H6 and H8 protons and the ribose H1’ and H2’ protons in the helical regions using the H6/H8(n) ↔ H1’(n) ↔ H6/H8(n+1) and H6/H8(n) ↔ H2’(n) ↔ H6/H8(n+1) connectivity pathways (Wüthrich, 1986). The ribose assignments were then confirmed and extended to the H3’ protons using through-bond couplings via a COSY or TOSCY experiment (data not shown). The connectivity pathway was broken at the abasic sites in the central region. Figure 7 shows the assignment pathway for the double helical region containing C_17_–A_23_. Complete assignments of the two helical regions are summarized in Supplementary Table S3.

The observed NOEs provide a number of results that are consistent with the presence the benzyl zipper motif. First, the magnitude of the NOE between the BndUH6 protons and the intranucleotide H1’ protons was large and consistent with a *syn* conformation for these nucleotides. In a *syn* conformation, the distance is ∼2.3 Å, compared to ∼3.8 Å in an *anti* conformation, and a much larger NOE is expected for the shorter distance due to the 1/r^6^ relationship (46). These large cross-peaks are labeled ‘Z8’ and ‘Z24’ in Figure 10. As shown in Figure 5, rotation of glycosidic bonds to the *syn* conformation rotates the benzyl groups toward the center of the helix. In addition, the chemical shifts of the benzyl protons (6.39–6.72 ppm) are upfield of what is observed for free nucleotides in solution (∼7.2 ppm, data not shown), suggesting that both rings are involved in stacking interactions with other aromatic rings.

Furthermore, the meta protons from each benzyl ring (6.66 and 6.72 ppm) show NOEs to the AH2 protons of the A-T base pairs above and below in the helix. The two AH2 protons (7.61 and 7.71 ppm) are near the center of the helix in an A-T base pair. It should be noted that two AH8 protons (8.04 and 8.14 ppm) also show NOEs the benzyl protons, suggesting a dynamic component to the structure. Because of the 1/r6 dependence of NOEs, conformational dynamics can allow observation of a collection of contacts that appear to be <5 Å, but are not possible with a single, static structure. Though the data were acquired 25°C below the melting temperature of the duplex (40°C versus 65°C, respectively), it is not unreasonable for the zipper region to be in fast exchange on the NMR time scale with some additional conformers.

The sum of these NMR results, however, provides strong evidence that the model duplex adopts a conformation with the benzyl rings stacked within the helix in fashion consistent with the zipper motif.

## CONCLUSION

The recent use of 5-N-carboxamide modified 2′-deoxyuridine nucleotides has dramatically increased the success of SELEX experiments with protein targets, resulting in both higher hit rates and better binding affinities. In order to understand the thermodynamic drivers that facilitate this selective advantage, we designed a series of experiments to understand the impact of these modifications to both DNA duplex stability and the stabilization of intramolecular single stranded structures.

With regard to duplex stability, the impact of the modifications was idiosyncratic. Within the constructs of two IL-6 SOMAmer sequences, only the bulky hydrophobic modifications, NapdU and 2-NapdU, were destablizing, and the most stabilizing modification was an aliphatic isobutyl moiety. The hydrophilic modifications such as MOEdU, IMdU and PyrdU were also stabilizing, but the overall effect was modest. Smaller aromatic groups such as BndU, PEdU and FBndU had a neutral or mildly stabilizing effect. Changes in thermal stability of the hybrid duplexes relative to DNA were the result of offsetting effects of enthalpy and entropy, with the enthalpic effect being dominant. The smaller aromatic groups such as BndU, PEdU and FBndU had a fairly neutral impact, with a net ‘cancellation’ of changes to ΔH° and ΔS°.

We next examined the impact of the modified nucleotides on the intramolecular folding of SOMAmers. Direct comparison of the melting temperatures of SOMAmers and their unmodified analogs showed that the modifications allow formation of more stable folded structures. It was interesting to note the NapdU nucleotide, which was destabilizing to duplex structures, allowed a more stable intramolecular structure to form. This suggests that outside the constraints of the double helix, which dominates stabilization when complementary strands are present, novel hydrophobic interactions can contribute to higher thermodynamic stability. In a similar fashion, within the benzyl zipper model compound, the stability provided by stacking benzyl rings drives the pyrimdine nucleotides into the normally less favorable *syn* conformation. Initial NMR data for the intramolecular structures suggest SOMAmers have defined structures at low temperature independent of their cognate proteins, and the transition cannot be adequately described by a two-state model.

Taken together, these observations suggest that modified nucleotides have an important impact on the thermodynamic stability of duplexes as well as on the folding of nucleic acid ligands.

## Supplementary Material

SUPPLEMENTARY DATA
