# Supplementary material for: Influence of 5-N-carboxamide modifications on the thermodynamic stability of oligonucleotides
Source: Nucleic Acids Res. 2015 Oct 4;43(19):9107–22. doi: 10.1093/nar/gkv981 (PMC4627095; doi:10.1093/nar/gkv981)
Supplement: SUPPLEMENTARY DATA [file supp_43_19_9107__index.html]

Influence of 5-N-carboxamide modifications on the thermodynamic stability of oligonucleotides — SUPPLEMENTARY DATA 

# Influence of 5-N-carboxamide modifications on the thermodynamic stability of oligonucleotides

## SUPPLEMENTARY DATA

- SUPPLEMENTARY DATA
