# Supplementary material for: Influence of 5-N-carboxamide modifications on the thermodynamic stability of oligonucleotides
Source: Nucleic Acids Res. 2015 Oct 4;43(19):9107–22. doi: 10.1093/nar/gkv981 (PMC4627095; doi:10.1093/nar/gkv981)
Supplement: SUPPLEMENTARY DATA [file supp_gkv981_Wolk-NAR-revised-manuscript-supplementary10-07-15.pdf]

SUPPLEMENTARY INFORMATION

Thermodynamic Parameters and the Two-State Model Approximation

Currently, there are four standard methods in the literature for determining thermodynamic parameters for oligonucleotides from measurements of absorbance at 260 nm vs. temperature (27). These include 1) analysis of T_m values as a function of concentration (lnc vs $1/T_m$), 2) analysis of lnK *vs.* 1/T within each melting curve, where K is the equilibrium constant for the DS $\leftrightarrow$SS transition, 3) obtaining $\Delta H°$ from the slope a plot of θ vs. temperature at the T_m, where θ = the fraction of strands in duplex form, and 4) a six parameter fit of each melting curve ($\Delta H°$, $\Delta S°$, and slope and intercept of the upper and lower baselines).

In principle, all four methods should yield the same results, and each has inherent strengths and weaknesses. For example, the lnK vs 1/T method is dependent on accurate estimations of upper and lower baselines, and obtaining $\Delta H°$ from the slope depends on excellent signal to noise through the transition phase. Similarly, the six parameter fit can lead to erroneous results when the baselines are not well defined. Therefore, it is prudent to use at least two of these methods to verify that the same results can be obtained. Toward this end, thermodynamic parameters ($\Delta H°$ and $\Delta S°$) were calculated using a van't Hoff approach (lnK *vs.* 1/T within each curve), and $\Delta H°$ was also calculated from $\Delta H° = 6RT_m^2(d\theta/dT)_{T=Tm}$, where $\Delta H°$ is enthalpy of the transition, R is the gas constant (1.987 cal/mol-K), T_m = the melting temperature, and θ = the fraction of strands in duplex form (28).

All four of these methods are based on the assumption of a two-state model, e.g., a transition from a homogeneous, temperature independent double stranded state to a homogeneous, temperature independent single stranded state. In reality, this is always an approximation, and the impact of the additional states on the extracted thermodynamic parameters depends, of course, on the both their abundance and the magnitude of the differences in energy from the core duplex and single stranded states. The errors are expected to get larger as the length of the oligonucleotides increases. This is due to both the increase in the variety of microstates available, as well the increased abundance of synthetic impurities.

For the longer oligonucleotides in this study (28-32 nucleotides), both issues are a valid concern. For example, high resolution UPLC methods and careful integration of chromatograms suggest that most synthetic oligonucleotides greater than 20 nucleotides are not more than about 80% pure, particularly those that contain modified nucleotides (data not shown). Because the majority of synthetic impurities are expected to form shorter and/or imperfect duplexes, these species will melt at lower temperatures and therefore create artifacts in the lower temperature portion of the melting curves. Impurities that are very similar in length to the parent sequence (e.g., n-1, n-2) will have very similar melting properties and cannot be fully isolated, though their thermodynamic values are much closer to those of the parent

duplex. Shorter impurities will cause greater deviation, and can typically be seen as deviations in the early stages of the transition region in the melting curves, most easily visualized in the derivative plots.

Longer duplexes are also more likely to form partially melted states, as well as a distribution of single stranded structures that can include folded structures with stacked and/or base-paired nucleotides. For example, the sigmoidal shapes of single stranded melts shown in Figure 1 indicate the presence of these additional states, though the population of these states is expected to be small when competing with a fully paired duplex structure. The deviation observed in the melting curves as a result of significant contributions of these additional states are likely to have an appearance similar to those caused by impurities, and these two possibilities cannot be distinguished from these data alone.

To ensure that the errors to the thermodynamic measurements caused by these deviations from a two-state model are small, the following approach was taken. First, agreement between the two methods used to determine $\Delta H°$, a standard van't Hoff approach (25, 26), and a slope method (28), was required. In addition, only the higher temperature portion of the transition region (e.g., $0.15 < \theta < 0.50$, where θ is the fraction of strands in duplex form) were used to determine the van't Hoff enthalpies. Puglisi and Tinoco (27) have recommended using the range ($0.15 < \theta < 0.85$) to avoid increasing error near the upper and lower baselines. Reducing the range to the region above the Tm reduces the contribution of both the impurity duplexes with T_ms significantly lower than the parent duplex and the contributions of folded, single stranded structures, which are likely to be two most significant deviations from the two state approximation. A similar approach was used by McTigue et al. (32), where deviation from two-state behavior was visualized clearly in the derivative plots, and this portion of the data set was omitted from a multi-parameter fit. They assigned the deviation from two-state behavior as "pre-melting" but it may contain a contribution from impurities. Second, the measured thermodynamic values were compared to those calculated from the sum of nearest neighbor contributions plus initiation and symmetry terms.

Method Details

Details of the two methods for determining the thermodynamic values are as follows. The first method utilized a standard van't Hoff approach (25, 26). For a two-state model, the equilibrium constant can be written as

$$K = \frac{2\theta}{(1-\theta)^2 C_T} = e^{-\Delta G°/RT} = e^{-\frac{\Delta H°}{RT} + -\frac{\Delta S°}{R}}$$

where:

θ = the fraction of strands in the double stranded state

C_T = the total concentration of all single strands.

At any temperature, θ can be obtained from

$$1 - \theta = \frac{A_{260}(T) - A_{260-DS}(T)}{A_{260-SS}(T) - A_{260-DS}(T)}$$

where:

$A_{260}(T)$ = the absorbance at 260 nm at temperature T

$A_{260\text{-}DS}(T)$ = the absorbance at 260 nm at temperature T for double stranded (lower) baseline

$A_{260\text{-}SS}(T)$ = the absorbance at 260 nm at temperature T for single stranded (upper) baseline

Standard enthalpies were also calculated from (28):

$\Delta H^\circ = 6RT_m^2(d\theta/dT)_{T=Tm}$

where:

ΔH° = enthpay of the transition

R = 1.987 cal/mol-K

T_m = the melting temperature

θ = the fraction of strands in duplex form

Linearity of the Van't Hoff Approach as a Function of the Range of θ

An example of the effect of reducing the temperature analysis range is shown in Figure S1, which shows the van't Hoff plot for a melting curve of the natural DNA duplex in series A. The black line represents a least squares fit of the set of points covering $0.15 < \theta < 0.85$. The red line represents a least squares fit of the range $0.15 < \theta < 0.50$. Both give excellent fits ($r^2 = 0.9975$ and 0.9990, respectively), but the slopes of the lines, which are proportional to ΔH°, differ by 9.6%. For the three repeats of each melt, the variation was smaller using the "late curve" approach (average CV = 2.9%, range = 1.0-7.1% for the full curve approach; average CV = 2.3%, range = 0.2-5.4% for the late curve approach). Consequently, this late curve analysis was used to determine the thermodynamic values in this report. It should be noted that the risk in using this approach is that factors such as sample evaporation and degradation during the high temperature portion of the experiment, if present, will distort the late portion of the curves.

Comparison of the van't Hoff Method and the Slope Method

Table S2 summarizes the ΔH° values calculated by the two methods for duplexes from both series. The differences range from 0.2-13.3%. In some cases, differences after the initial calculations were larger. As mentioned above, the source of the differences is most likely the choice of baselines used for the van't Hoff analysis (27). In cases where the agreement was poor, the fits to define the upper baselines were scrutinized carefully, since the high melting temperature of some of the duplexes resulted in a limited

temperature range from which to determine the slope. Alternative choices were tested for agreement with the slope method as well “reasonableness” based on visual appearance. The results in Table S2 represent the values obtained after these adjustments.

Comparison of Measured and Calculated Thermodynamic Values

As an additional check on the accuracy of the measured thermodynamic parameters and the validity for the two-state model for these calculations, a comparison was made to values calculated from the sum of nearest-neighbor interactions plus initiation and symmetry terms (28, 47, 48, 49). Nearest neighbor interactions were determined from sets of short, model oligonucleotide duplexes where the two state model was assumed to be a reasonable approximation. Using software developed by Owczarzy and co-workers (version 1.02, 2015 (47)), values were calculated for the three unmodified duplexes in this study, and compared to the measured values. These data are shown in Table S3. Differences between measured and calculated values ranged from (0.7 to 7.5%), suggesting that the two-state approximation did not result in large errors in the measured values.

Comparison of Thermodynamic Values in Series A and Series B

Though the consistency checks described above suggest that the errors in the thermodynamic measurements are small, comparison of values were made within each series only. The similarity of the duplexes within each series indicates that errors will be systematic, allowing meaningful comparisons.

Tables S4 and S5 present the thermodynamic parameters determined for the subset of sequences analyzed for the two series, listed in order of increasing T_m. Table S4b and S5b show the changes relative to the unmodified DNA duplex in each series. The values clearly indicate that the changes in duplex stability caused by the presence of the modified nucleotides are driven by opposing contributions from enthalpy and entropy terms, with enthalpy being the dominant term. For example, the duplex with the lowest T_m value within series A is the 8-Nap variant, which melts about 8 °C lower than the unmodified duplex. The $\Delta H°$ for duplex formation is -193 kcal/mol, 55 kcal/mol less favorable than the unmodified variant. In contrast, the $-T\Delta S°$ term, which is expected to be unfavorable for two strands coming together, is 158 kcal/mol, which is 42.9 kcal/mol more favorable (e.g., less unfavorable) than the all-DNA helix. In other words, only the enthalpic term drives toward lower stability, and dominates the entropic term. In contrast, the duplex with the highest T_m value in this series is the 8-iBudU variant, which melts 4 °C higher than the unmodified duplex. The $\Delta H°$ for duplex formation is -278 kcal/mol, 30 kcal/mol more favorable than the all-DNA analog. In contrast, the $-T\Delta S$ term, is 223 kcal/mol, or 30 kcal/mol less favorable (e.g., more unfavorable) than the unmodified duplex. Again, only the enthalpic term drives toward higher stability, and dominates the entropic term. This same pattern is observed within series B, although the magnitude of the effect is smaller (Table S5 and Figure S3).

SUPPLEMENTARY REFERENCES

47. Owczarzy,R., You,Y., Groth,C.L. and Tatarov,A.V. (2011) Stability and Mismatch Discrimination of Locked Nucleic Acid−DNA Duplexes. *Biochemistry*, **50**, 9352−9367.

48. SantaLucia, J. Jr. (1998) A unified view of polymer, dumbbell, and oligonucleotide DNA nearest-neighbor thermodynamics. *Proc. Natl. Acad. Sci. U.S.A.*, **95**, 1460−1465.

49. Sugimoto, N., Nakano, S., Yoneyama, M. and Honda, K. (1996) Improved thermodynamic parameters and helix initiation factor to predict stability of DNA duplexes. *Nucleic Acids Res.*, **24(22)**, 4501-4505.

SUPPLEMENTARY FIGURES AND TABLES

Table S1. Summary of the effect of hydrophilic modifications on duplex T_m values.

Table S2. Comparison of $\Delta H°$ values obtained by the van't Hoff and $d\theta/dT$ methods.

Table S3. Comparison of measured and calculated (Owczarzy and co-workers (software version 1.02, 2015 (45)) thermodynamic values for three unmodified duplexes.

Table S4. a) Thermodynamic parameters for formation of oligonucleotide duplexes from series A. Parameters were obtained from van't Hoff analyses and $d\theta/dT$ analyses of optical melting at 260 nm in high salt (1 M NaCl, 50 mM sodium phosphate, pH 7.4). b) Values relative to the standard DNA control.

Table S5. a) Thermodynamic parameters for formation of oligonucleotide duplexes from series B. Parameters were obtained via from van't Hoff analyses of optical melting at 260 nm in high salt (1 M NaCl, 50 mM sodium phosphate, pH 7.4). b) Values relative to the standard DNA control.

Table S6. Summary of chemical shift assignments in the ^1H NMR spectrum of the proposed Benzyl Zipper duplex (0.7 mM duplex in 130 mM NaCl, 20 mM Na-Phos, 1 mM EDTA, pH 7, 40 °C).

Figure S1. Example melting curves (based on absorbance at 260 nm) for oligonucleotide duplexes from series A and series B (8-NapdU, 8-BndU, and 8-dT variants) in high salt (1 M NaCl, 50 mM sodium phosphate, pH 7.4). Each curve is normalized to an absorbance of 1.0 at 95 °C.

Figure S2. Example van't Hoff plot (lnK *vs.* 1/dT) for a melting curve at 260 nm for the unmodified duplex from series A in high salt (1 M NaCl, 50 mM sodium phosphate, pH 7.4). Comparison of least squares fit of the data for $0.15 < \theta < 0.85$ (black) *vs.* $0.50 < \theta < 0.85$ (red).

Table S1. Summary of the effect of hydrophilic modifications on duplex T_ms.

	Series A Tms (°C)					Series B Tms (°C)			
**MOD**	**high salt**	**Δ from dT**	**low salt**	**Δ from dT**		**high salt**	**Δ from dT**	**low salt**	**Δ from dT**
dT	80.7	-	72.9			83.1	-	75.6	-
BndU	83.0	2.3	75.0			85.4	2.3	77.4	1.8
MOEdU	82.3	1.6	74.7			85.0	1.9	78.9	3.3
ImdU	82.6	1.9	75.6			85.6	2.4	78.6	3.0
PyrdU	83.3	2.6	75.9			84.0	0.9	78.1	2.5
average of hydrophilic mods		2.0	2.5				1.8		2.3

Table S2. Comparison of ΔH° values obtained by the van't Hoff and dθ/dT methods.

Series A

	ΔH_{VH} (kcal/mol)	ΔH_{sI} (kcal/mol)	% difference
8-NapdU	-193	-182	6.0
8-2NapdU	-201	-201	-0.2
8-TrpdU	-230	-217	5.9
dT	-248	-227	9.4
7-BndU/1-NapdU	-251	-231	8.5
8-BndU	-248	-227	9.2
8-PyrdU	-245	-225	8.8
8-iBudU	-278	-278	0.0

Series B

	ΔH_{VH} (kcal/mol)	ΔH_{sI} (kcal/mol)	% difference
6-NapdU	-178	-171	3.9
6-2NapdU	-183	-183	-0.2
6-TrpdU	-211	-187	13.3
dT	-229	-224	2.6
6-BndU	-229	-222	3.1
6-PyrdU	-238	-221	7.9
6-iBudU	-269	-251	7.1

Table S3. Comparison of measured and calculated (Owczarzy and co-workers (software version 1.02, 2015 (45)) thermodynamic values for three unmodified duplexes.

unmodified duplex	ΔH° (kcal/mol)	ΔS° (cal/mol·K)	ΔG°_{25} (kcal/mol)	ΔG°_{37} (kcal/mol)	Tm (°C) Q=0.5	Tm (°C) $(dA_{260}/dT)_{max}$
Series A						
measured values	-248	-674	-46.9	-38.9	80.4	81.3
calculated values	-255.60	-692.93	-49.0	-40.69	81.90	81.90
% difference	-3.0	-2.7	-4.2	-4.5	-1.8	-0.7
Series B						
measured values	-229	-615	-45.6	-38.3	83.0	83.5
calculated values	-226.00	-604.15	-45.87	-38.62	84.91	84.91
% difference	1.3	1.8	-0.5	-0.9	-2.2	-1.7
Benzyl zipper series						
measured values	-140	-373	-28.8	-24.3	75.6	75.6
calculated values	-131.30	-346.93	-27.86	-23.70	79.21	79.21
% difference	6.6	7.5	3.3	2.6	-4.6	-4.6

Table S4. a) Thermodynamic parameters for formation of oligonucleotide duplexes from series A. Parameters were obtained from van't Hoff analyses and dθ/dT analyses of optical melting at 260 nm in high salt (1 M NaCl, 50 mM sodium phosphate, pH 7.4). b) Values relative to the standard DNA control.

a)

		$\Delta H°$ (kcal/mol)	$-T\Delta S°$ (kcal/mol)	$\Delta G°_{25}$ (kcal/mol)	Tm (°C)	
					Q=0.5	$(dA_{260}/dT)_{max}$
↑	8-NapdU	-193	158	-34.9	72.3	73.1
	8-2NapdU	-201	164	-36.3	73.0	73.6
destabilized	8-TrpdU	-230	186	-43.7	79.0	79.5
	dT	-248	201	-47.4	80.4	81.3
	7-BndU/1NapdU	-251	203	-48.0	80.7	81.3
stabilized	8-BndU	-248	200	-48.3	82.1	83.0
	8-PyrdU	-245	197	-48.2	82.7	83.6
↓	8-iBudU	-278	223	-54.5	84.3	84.6

b)

	$\Delta\Delta H°$ (kcal/mol)	$\Delta(-T\Delta S°)$ (kcal/mol)	$\Delta\Delta G°_{25}$ (kcal/mol)	ΔTm (°C)	
				Q=0.5	$(dA_{260}/dT)_{max}$
8-NapdU	55	-43	12.5	-8.1	-8.2
8-2NapdU	47	-37	11.1	-7.4	-7.7
8-TrpdU	18	-15	3.7	-1.4	-1.8
dT	0	0	0.0	0.0	0.0
7-BndU/1NapdU	-3	2	-0.6	0.3	0.0
8-BndU	0	-1	-0.9	1.7	1.7
8-PyrdU	3	-4	-0.8	2.3	2.3
8-iBudU	-30	22	-7.1	3.9	3.3

Table S5. a) Thermodynamic parameters for formation of oligonucleotide duplexes from series B. Parameters were obtained from van't Hoff analyses of optical melting at 260 nm in high salt (1 M NaCl, 50 mM sodium phosphate, pH 7.4). b) Values relative to the standard DNA control.

a)

		$\Delta H°$ (kcal/mol)	$-T\Delta S°$ (kcal/mol)	$\Delta G°_{25}$ (kcal/mol)		Tm (°C) Q=0.5	Tm (°C) $(dA_{260}/dT)_{max}$
	6-NapdU	-178	142	-36		79.9	80.9
	6-2NapdU	-183	146	-37		80.5	81.4
**destabilized**	6-TrpdU	-211	184	-46		83.1	83.8
	dT	-229	184	-43		83.0	83.5
**stabilized**	6-BndU	-229	182	-46		84.4	84.9
	6-PyrdU	-238	190	-48		84.9	85.8
	6-iBudU	-269	215	-54		85.9	86.2

b)

	$\Delta\Delta H°$ (kcal/mol)	$\Delta(-T\Delta S°)$ (kcal/mol)	$\Delta\Delta G°_{25}$ (kcal/mol)		ΔTm (°C) Q=0.5	ΔTm (°C) $(dA_{260}/dT)_{max}$
6-NapdU	52	-42	6.8		-3.1	-2.6
6-2NapdU	47	-38	5.7		-2.5	-2.1
6-TrpdU	18	0	-2.9		0.1	0.3
dT	0	0	0.0		0.0	0.0
6-BndU	1	-1	-3.5		1.4	1.4
6-PyrdU	-9	6	-5.4		1.9	2.3
6-iBudU	-39	31	-11.1		2.9	2.7

Table S6. Summary of chemical shift assignments in the ^{1}H NMR spectrum of the proposed Benzyl Zipper duplex (0.7 mM duplex in 130 mM NaCl, 20 mM Na-Phos, 1 mM EDTA, pH 7, 40 °C).

	H6/H8	H5	H1'	H2"	H2'	H3'
**G1**	8.046		6.059	2.85	2.70	4.91
**C2**	7.577	5.46	6.147	2.56	2.23	4.94
**C3**	7.508	5.659	5.666	2.47	2.13	4.90
**G4**	7.961		5.424	2.76	2.71	5.04
**C5**	7.382	5.43	5.943	2.38	1.99	4.83
**C6**	7.433	5.69	5.250	2.15	1.93	4.81
**A7**	8.137		5.912	2.75	2.65	
**Z8**	7.861		5.845	2.65	2.53	4.89
**D9**						
**T10**	7.510		5.775	2.57	2.04	
**C11**	7.634	5.78	5.935	2.45	2.25	4.91
**C12**	7.510	5.701	5.571	2.43	2.12	4.90
**G13**	7.978		5.956	2.80	2.75	5.05
**C14**	7.448	5.46	6.018	2.48	2.08	4.87
**C15**	7.528	5.74	5.690	2.38	2.05	4.88
**G16**	8.022		6.243	2.70	2.44	4.75

	H6/H8	H5	H1'	H2"	H2'	H3'
**C32**	7.490	5.496	6.235	2.26	2.22	4.57
**G31**	7.812		6.033	2.80	2.60	5.01
**G30**	7.897		5.673	2.81	2.73	5.04
**C29**	7.313	5.358	5.715	2.38	1.93	4.88
**G28**	7.835		5.921	2.76	2.64	
**G27**	7.955		5.522	2.78	2.70	5.02
**T26**	7.366		5.610	2.23	1.73	4.84
**D25**						
**Z24**	7.813		5.804	2.64	2.55	
**A23**	8.038		5.916	2.75	2.64	5.06
**G22**	7.715		5.485	2.60	2.51	5.00
**G21**	7.838		5.388	2.67	2.63	5.07
**C20**	7.283	5.372	5.710	2.29	1.86	4.87
**G19**	7.882		5.992	2.79	2.66	
**G18**	7.988		5.575	2.82	2.77	5.04
**C17**	7.658	5.970	5.808	2.43	1.93	4.75

D spacer assigments

H1'	H1"	H2'	H2"
4.14	4.06	2.32	2.22
4.13	4.04	2.29	2.20

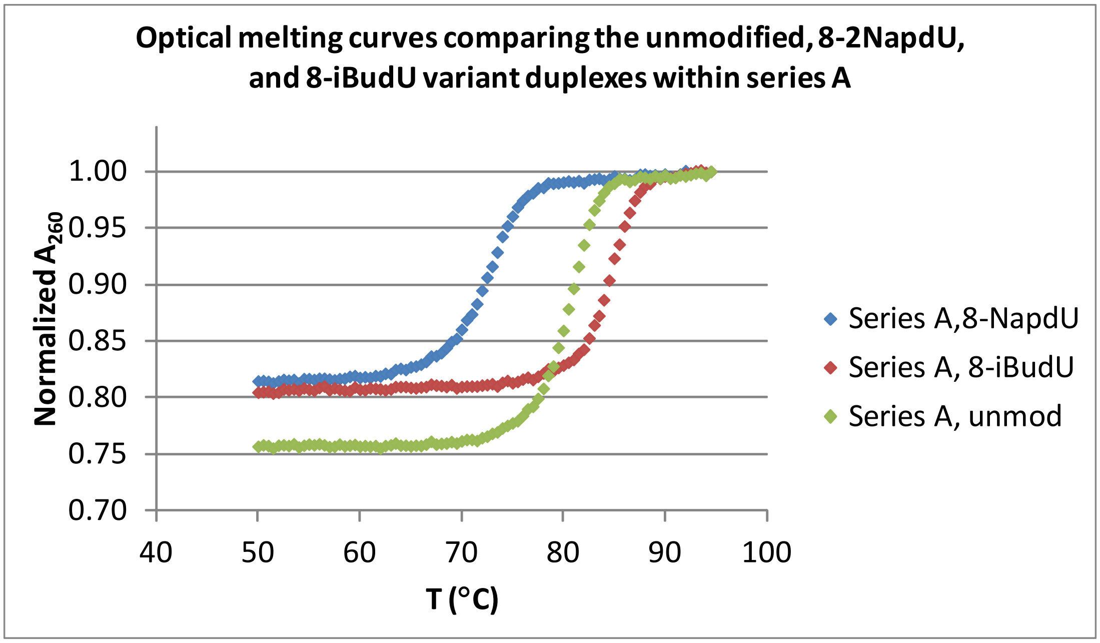

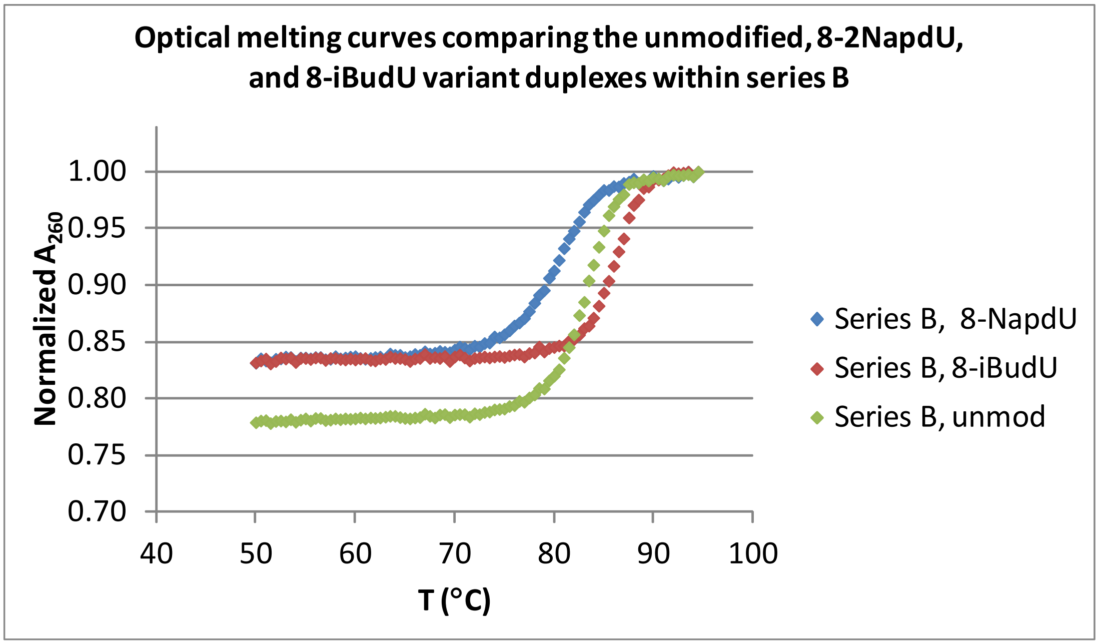

Figure S1. Example melting curves (based on absorbance at 260 nm) for oligonucleotide duplexes from series A and series B (8-NapdU, 8-BndU, and 8-dT variants) in high salt (1 M NaCl, 50 mM sodium phosphate, pH 7.4). Each curve is normalized to an absorbance of 1.0 at 95 °C.

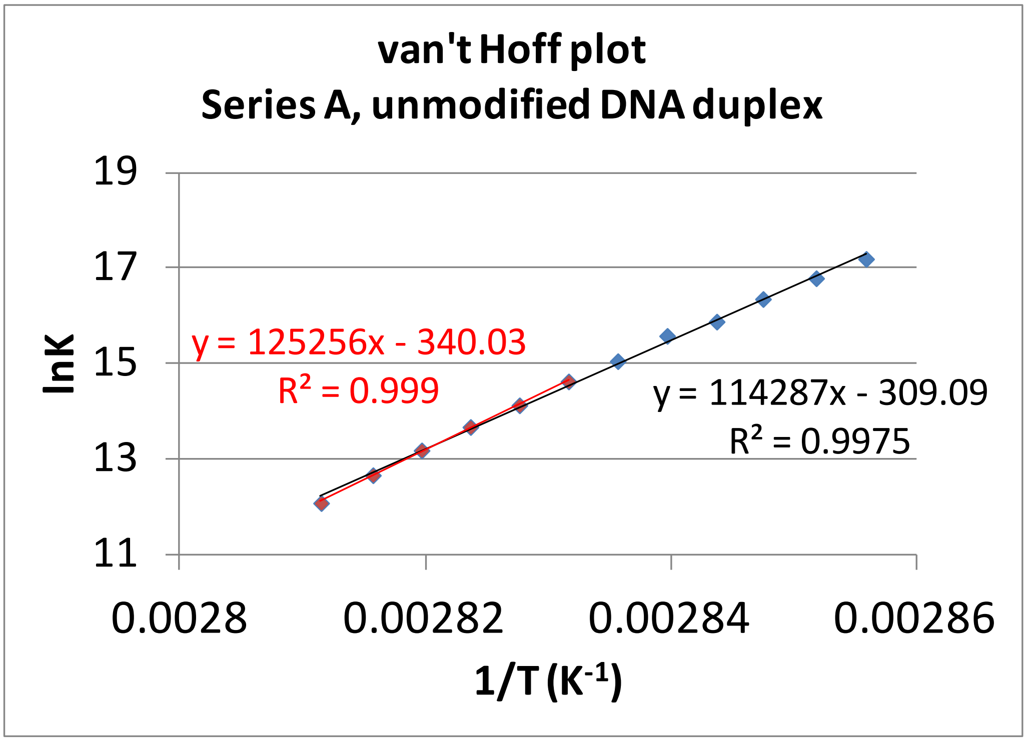

Figure S2. Example van't Hoff plot (lnK *vs.* 1/T) for a melting curve at 260 nm for the unmodified duplex from series A in high salt (1 M NaCl, 50 mM sodium phosphate, pH 7.4). Comparison of least squares fit of the data for $0.15 < \theta < 0.85$ (black) *vs.* $0.50 < \theta < 0.85$ (red).

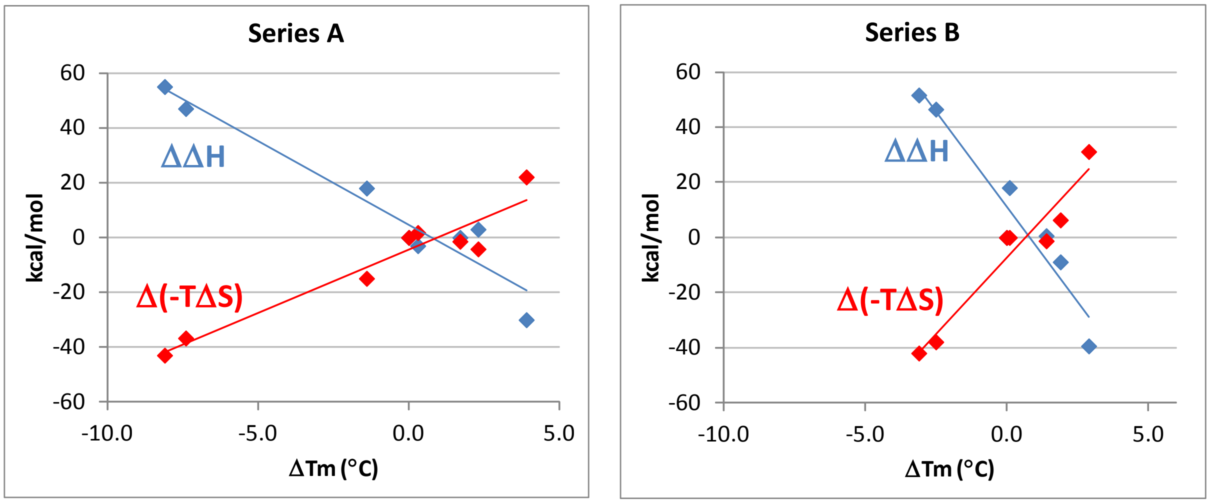

Figure S3. Comparison of ΔΔH° or Δ(-TΔS°) values for series A and series B as a function of the change in T_m for the various duplex sequences compared to the unmodified analogs. (based on the van't Hoff analyses of optical melting at 260 nm in high salt (1 M NaCl, 50 mM sodium phosphate, pH 7.4).

a)

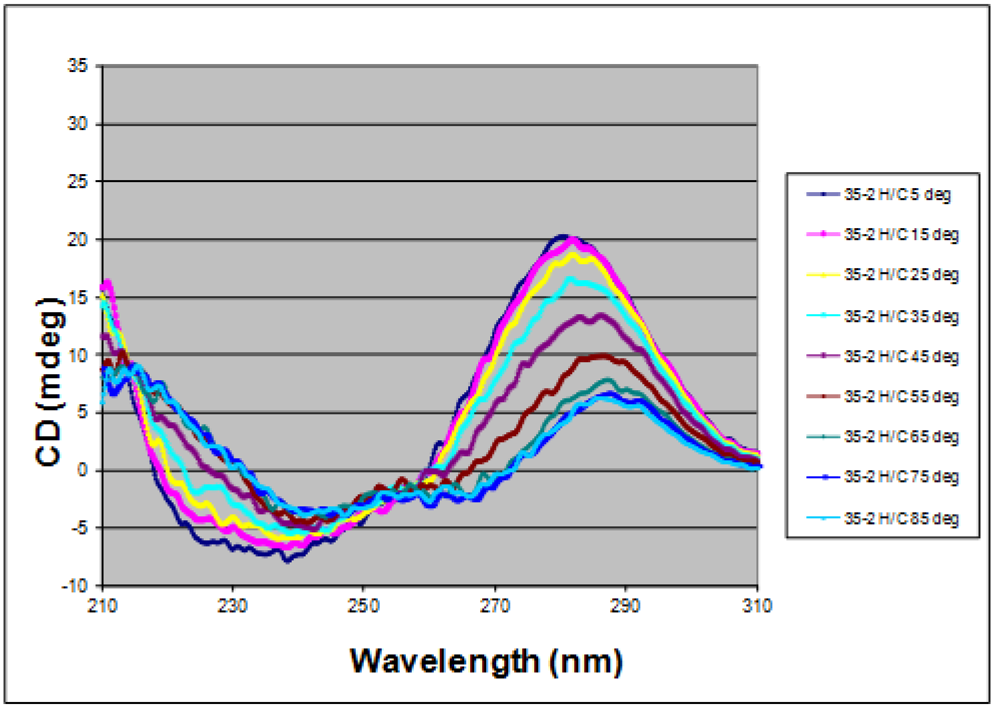

b)

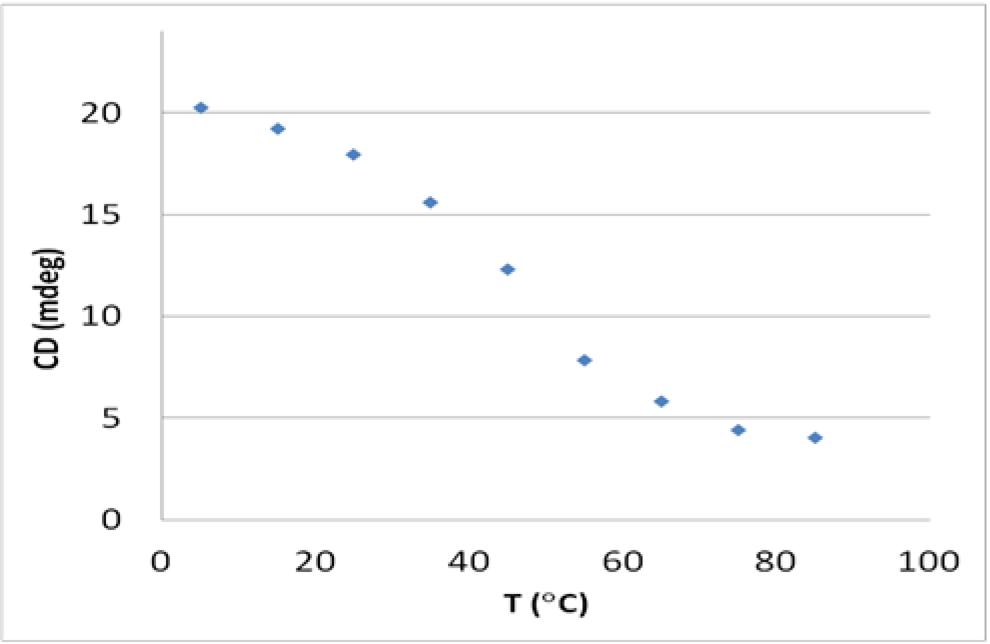

Figure S4. a) CD spectra of the NGF-β SOMAmer reagent SL1047 as a function of temperature (0.1 mM SOMAmer, 130 mM NaCl, 20 mM Na-phos, 1 mM EDTA, pH 7. b) CD intensity at 280 nm as a function of temperature.
